# Novel Reassortant Avian Influenza A(H5N1) Virus in Human, Southern Vietnam, 2014

**DOI:** 10.3201/eid2203.151360

**Published:** 2016-03

**Authors:** Ikuyo Takayama, Nguyen Trung Hieu, Masayuki Shirakura, Mina Nakauchi, Seiichiro Fujisaki, Hitoshi Takahashi, Shiho Nagata, Nguyen Thanh Long, Takato Odagiri, Masato Tashiro, Tsutomu Kageyama

**Affiliations:** National Institute of Infectious Diseases, Tokyo, Japan (I. Takayama, M. Shirakura, M. Nakauchi, S. Fujisaki, H. Takahashi, S. Nagata, T. Odagiri, M. Tashiro, T. Kageyama);; Pasteur Institute, Ho Chi Minh City, Vietnam (N.T. Hieu, N.T. Long)

**Keywords:** influenza, avian influenza H5N1 virus, phylogenetic analysis, reassortment, reassortant viruses, Vietnam, poultry, influenza in birds, hemagglutinins, viruses, zoonoses

**To the Editor:** The first case of human infection with highly pathogenic avian influenza A(H5N1) virus in Vietnam was reported in December 2003 (*1*), and >120 human cases were confirmed through 2013, with a high case-fatality rate (*2*). In 2013, clade 2.3.2.1a/c H5N1 viruses circulated widely in poultry across the country, although clade 1.1.1/1.1.2 H5N1 viruses predominated in poultry from the Mekong Delta region to central Vietnam (*3*,*4*). 

In 2014, two cases of human infection with A(H5N1) virus were identified in southern Vietnam. One case was associated with a clade 1.1.2 reassortant virus, A/Vietnam/14012902/2014 (Global Initiative on Sharing All Influenza Data [GISAID; http://www.gisaid.org] accession nos. EPI624919–EPI624926), which had been previously detected in Cambodia and Vietnam (*5*,*6*). We isolated the virus from the other case, performed phylogenetic analysis to identify the clade of this virus, and identified a novel virus that had undergone gene reassortment. 

The case-patient was a 52-year-old man who lived in Binh Phuoc Province (140 km northeast of Ho Chi Minh City). On January 11, 2014, he experienced mild fever and general fatigue; high fever developed on January 13. He was hospitalized with dyspnea on January 16 and died 2 days later. He was not given antiviral drug treatment. Dead poultry infected with H5N1 viruses were found scattered near his house during January 1–16, and he buried his 2 dead chickens on January 5. H5N1 virus infection was detected in the patient’s throat swab specimen by real-time reverse transcription PCR at the Pasteur Institute in Ho Chi Minh City. Virus was isolated by inoculating the throat swab specimens into 10-day-old embryonated chicken eggs; the resulting isolate, A/Vietnam/14011801/2014 (GISAID accession nos. EPI624911–EPI624918), then underwent gene sequencing. The 8 viral genes were amplified with SuperScript III Reverse Transcriptase Kit (Fisher Scientific, Pittsburg, PA, USA) and Phusion High-Fidelity DNA Polymerase (New England BioLabs, Ipswich, MA, USA) with specific paired primers, according to the manufacturer’s instructions, and sequenced on an ABI 3730 automated sequencer with Big-Dye Terminator Cycle Sequencing reagents (Applied Biosystems, Foster City, CA, USA). Whole genome sequence was determined.

By gene sequencing analysis, A/Vietnam/14011801/2014 was found to have the multibasic cleavage site of hemagglutinin (HA) protein, which indicates highly pathogenic avian influenza A(H5N1) viruses, and was shown to predict binding specificity to an avian α2,3 sialic acid receptor. The neuraminidase gene possessed no amino acid substitutions associated with decreased antiviral activity, nor did the virus have amino acid substitutions associated with increased adaptation, virulence, infectivity, or transmissibility in mammalian hosts, including the E627K and D701N mutations in polymerase basic protein 2 (*7*).

Phylogenetic analyses of the 8 viral genes of A/Vietnam/14011801/2014 were performed by using databases (GISAID and the Influenza Virus Resource, National Center for Biotechnology Information, Bethesda, MD, USA; http://www.ncbi.nlm.nih.gov/genomes/FLU/FLU.html) that contained complete sequences of viral genomes belonging to clades 1.1.1, 1.1.2, and 2.3.2.1 a/b/c, most of which were collected in Vietnam, particularly after 2012. Neighbor-joining and Kimura 2-parameter methods were implemented by using MEGA version 5.0 software (http://www.megasoftware.net). Reliability of the phylogenetic analysis was tested by using 1,000 bootstrap replications. Lineages of the HA gene were defined by using previously described criteria (*8*). Lineages of the other 7 genes were defined by using criteria and nomenclature of Nguyen et al. (*9*).

The HA of A/Vietnam/14011801/2014 belonged to clade 2.3.2.1c (online Technical Appendix Figure, panel A, http://wwwnc.cdc.gov/EID/article/23/3/15-1360-Techapp.pdf). The neuraminidase, polymerase basic proteins 1 and 2, and polymerase acid protein genes of this virus were also derived from respective lineages of ancestor clade 2.3.2.1c (Technical Appendix Figure, panels B–E). However, nucleoprotein, matrix, and nonstructural genes were classified as lineages of ancestor clade 2.3.2.1a (online Technical Appendix Figure, panels F–H) and differed from the gene lineages of almost all clade 2.3.2.1c viruses isolated from poultry in Vietnam. As reported in the Influenza Virus Resource, 2 viruses collected in Vietnam in December 2013 (A/muscovy duck/Long An/43/2013 and A/muscovy duck/Long An/46/2013) were similar reassortant viruses of clade 2.3.2.1c (Figure). However, the ancestor of the nonstructural gene lineage of these 2 viruses is clade 2.3.2.1c, which differs from A/Vietnam/14011801/2014. The differences indicate that A/Vietnam/14011801/2014 is a novel reassortant virus between clades 2.3.2.1a and 2.3.2.1c, between clades 1.1.2 and 2.3.2.1c, or both (Figure). This novel reassortant virus has not been reported in poultry in Vietnam, although novel reassortants between clade 1.1.2 and clade 2.3.2.1a viruses have been detected in Vietnam since 2013 (i.e., A/Vietnam/VP13-28H/2013, GISAID accession nos. EPI624927–EPI624934; and A/Vietnam/14012902/2014) (*6*). These novel reassortment viruses were first identified in human, animal, and environmental samples in Cambodia in 2013 (*5*). Other novel gene reassortments in clade 2.3.2.1 viruses have been previously reported (*10*), and new clade 2.3.4.4 viruses have been observed in Vietnam since 2014.

**Figure:**
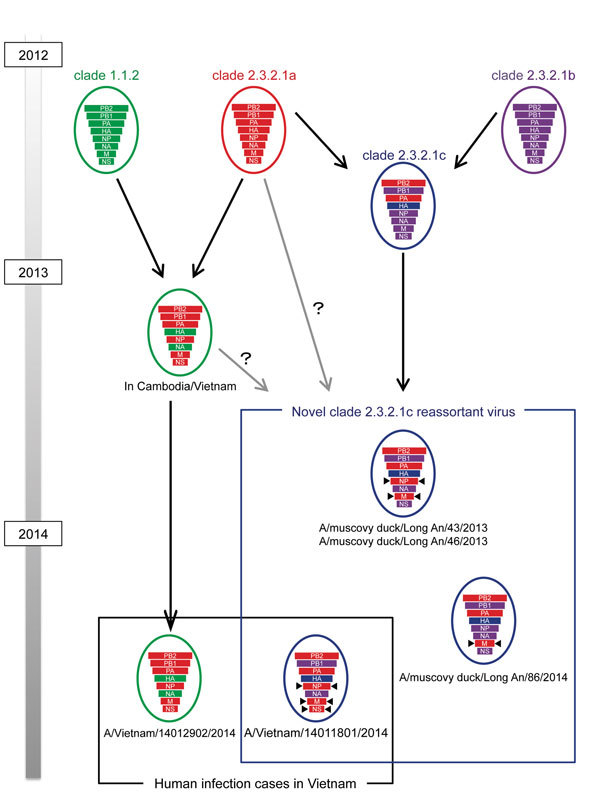
Novel reassortant virus (A/Vietnam/14011801/2014) identified in a human case of influenza A(H5N1) virus infection in Vietnam, 2014. Ancestry of genes is denoted in the hemagglutinin clades. Arrows indicate genes that differ from the gene lineages of original clade 2.3.2.1c viruses. Colors indicate ancestry of each gene: green, clade 1.1; red, clade 2.3.2.1a; purple, 2.3.2.1b; and blue, clade 2.3.2.1c.

As multiple clade viruses co-circulate, reassortment events occur frequently in Vietnam. Continuous surveillance of avian influenza A(H5N1) viruses, not only in humans but also in poultry and wild birds, is needed for infection control measures during epidemics of these viruses.

**Technical Appendix.** Phylogenetic analyses of the genes of the avian influenza A(H5N1) virus identified in a human in Vietnam, 2014.
