# Supplementary material for: Novel Reassortant Avian Influenza A(H5N1) Virus in Human, Southern Vietnam, 2014
Source: Emerg Infect Dis. 2016 Mar;22(3):557–9. doi: 10.3201/eid2203.151360 (PMC4766877; doi:10.3201/eid2203.151360)
Supplement: Supplementary file 1 — Technical Appendix. Phylogenetic analyses of the genes of the avian influenza A(H5N1) virus identified in a human in Vietnam, 2014. [file 15-1360-Techapp-s1.pdf]

Article DOI: http://dx.doi.org/10.3201/eid2203.151360

Novel Reassortant Avian Influenza A(H5N1) Virus in Human, Southern Vietnam, 2014

Technical Appendix

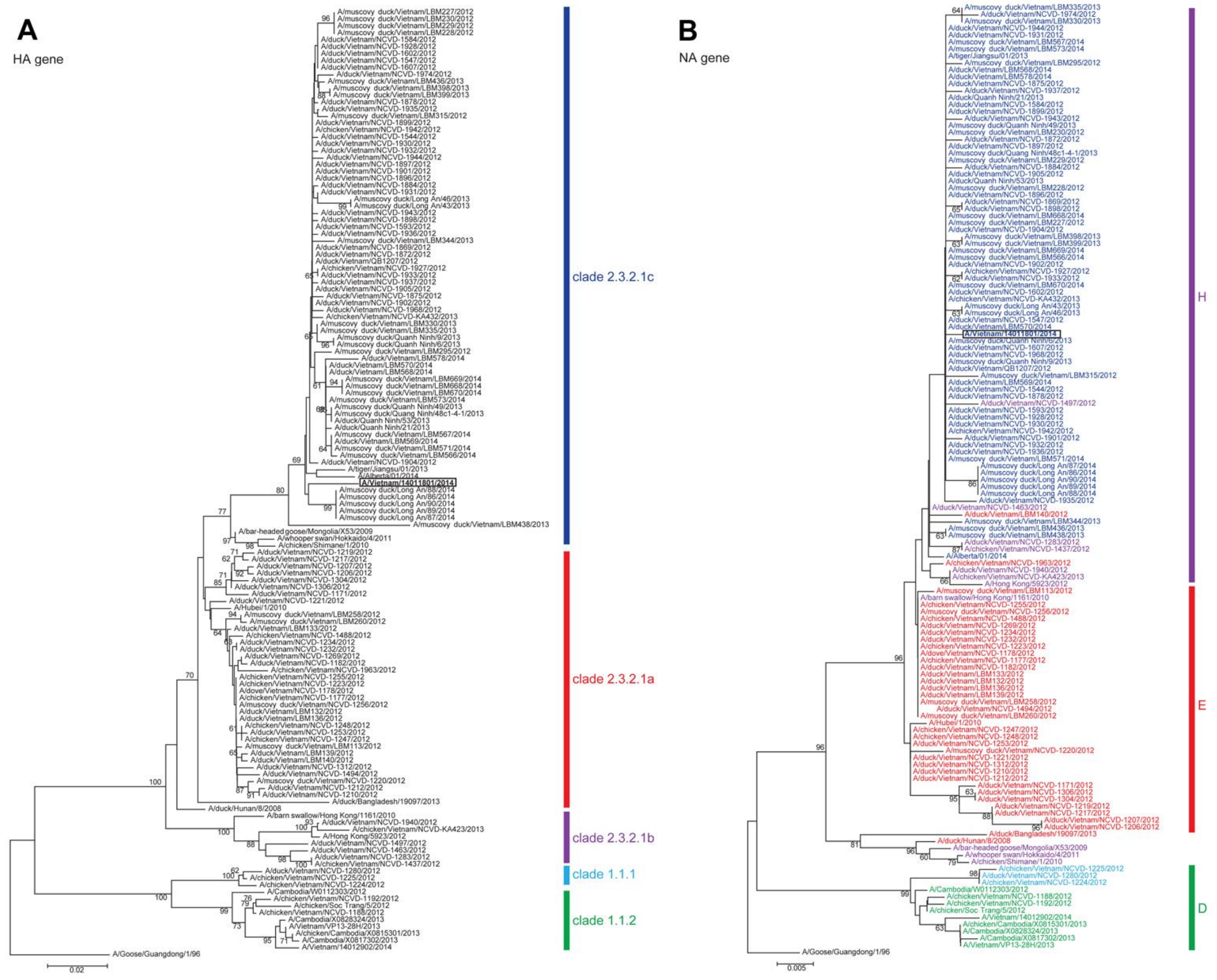

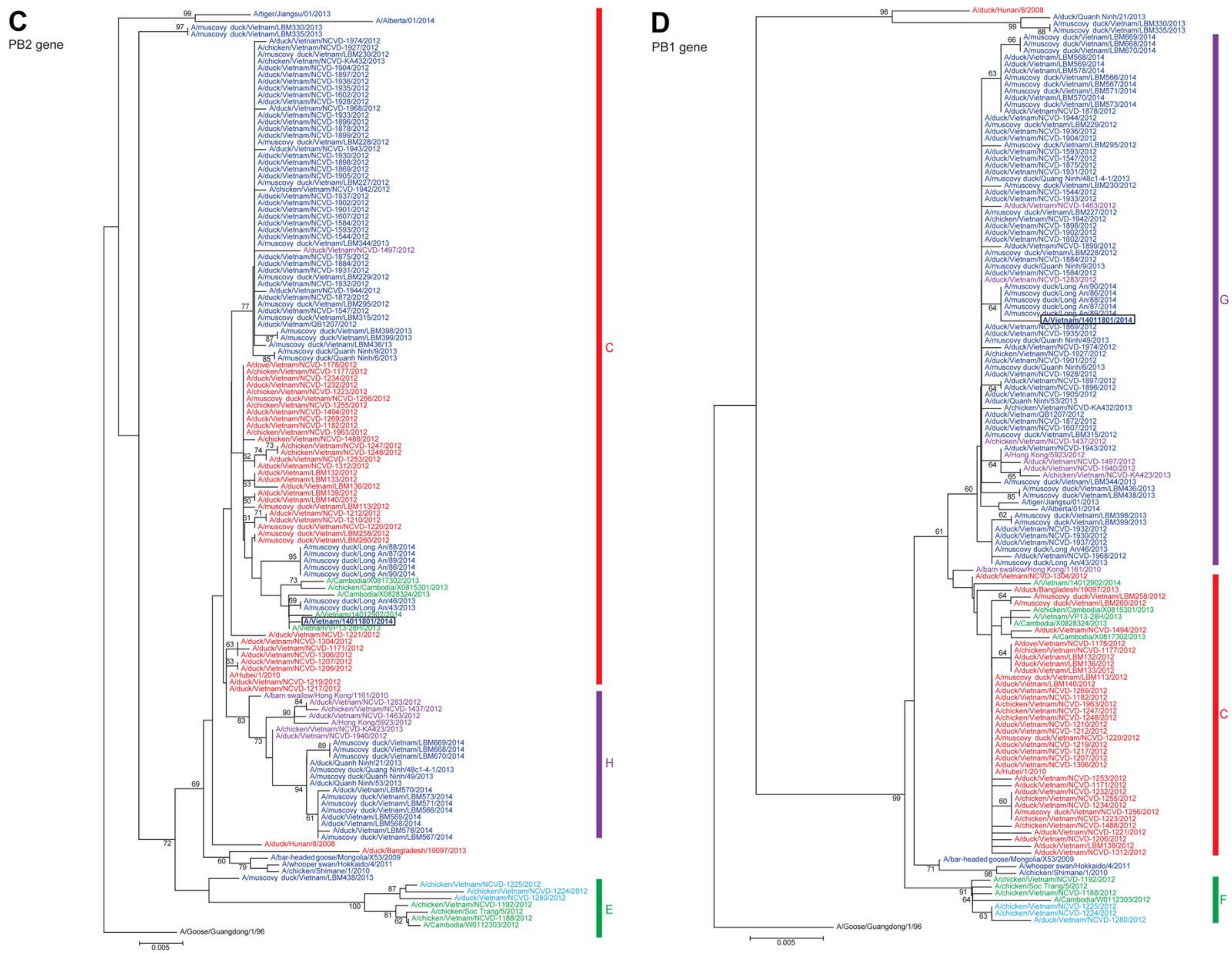

C
PB2 gene
D
PB1 gene

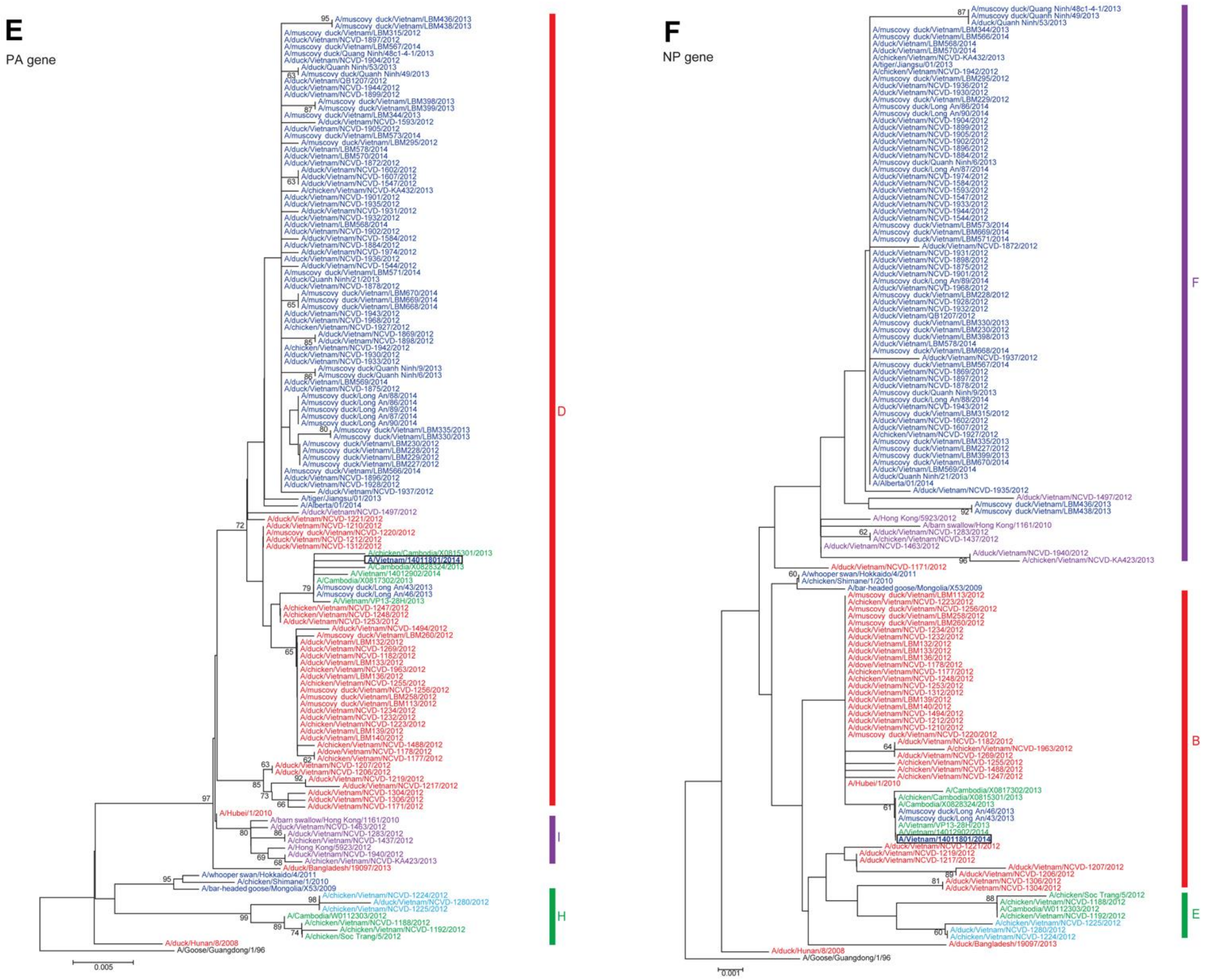
E
PA gene
D
I
H
0.005
F
NP gene
F
B
E
0.001

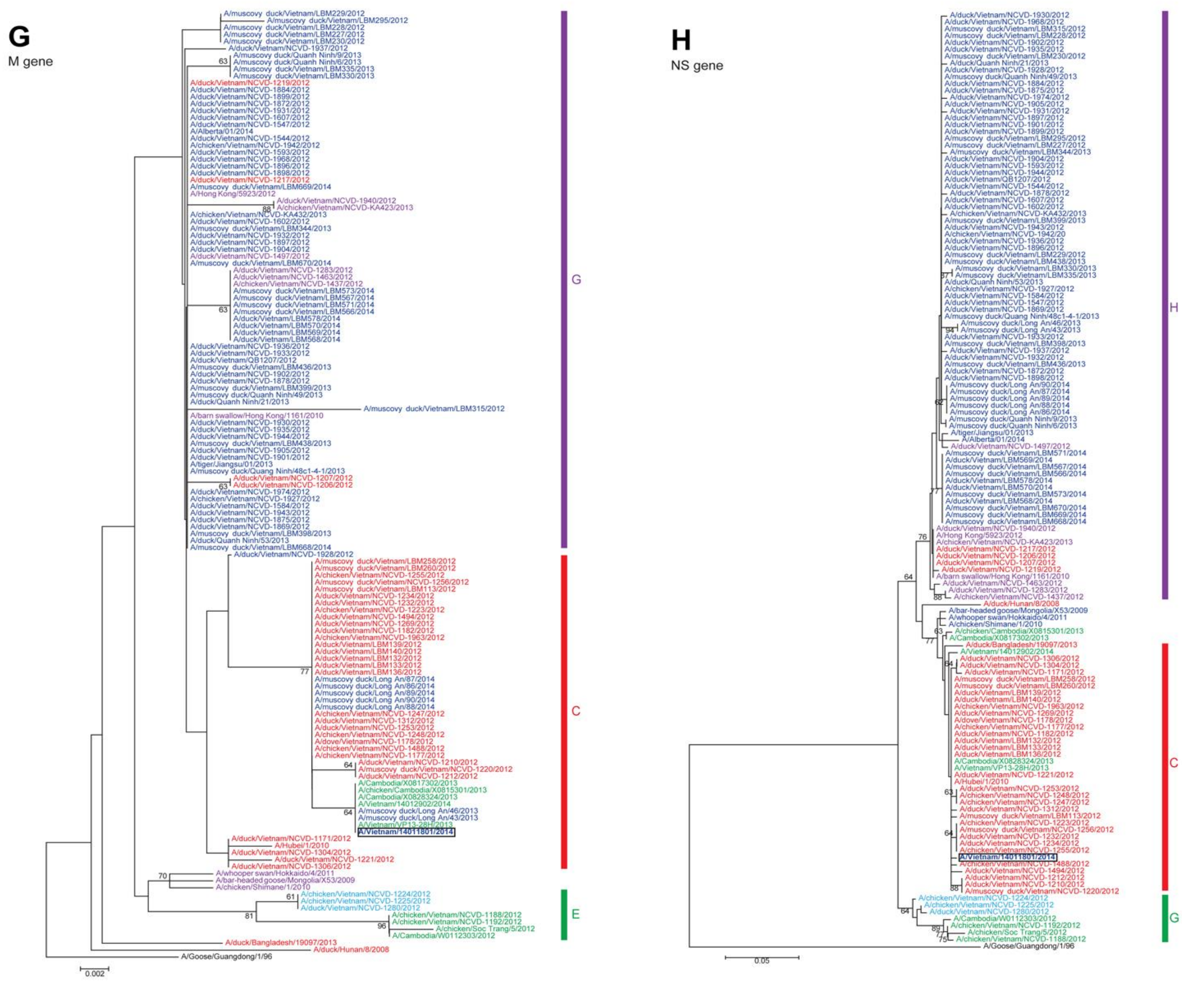

Technical Appendix Figure. Phylogenetic analyses of the genes of the avian influenza A(H5N1) virus described in this study: A) hemagglutinin (HA) gene; B) neuraminidase (NA) gene; C) polymerase basic (PB) 2 gene; D) PB1 gene; E) polymerase acid (PA) protein gene; F) nucleoprotein (NP) gene; G) matrix (M) gene; and H) nonstructural (NS) gene. Trees were constructed by using the neighbor-joining method. The evolutionary distances were computed by using the Kimura 2–parameter method. Bootstrap values were calculated from 1,000 replicates and values >60% are shown next to branches. Sequences of the A(H5N1) virus isolated in this study are in bold font and inside a box. Viruses are colored on the basis of their hemagglutinin clade: 1.1.1 in light blue; 1.1.2 in green; 2.3.2.1a in red; 2.3.2.1b in purple; and 2.3.2.1c in dark blue. Scale bars indicate nucleotide substitutions per site.
